# Effects of a multifaceted quality improvement intervention in reducing mortality and bloodstream infection in ICUs: insights from the QUALITI initiative

**DOI:** 10.1186/cc10672

**Published:** 2012-03-20

**Authors:** AB Cavalcanti, JC Othero, JC Mouro, KN Silva, ES Victor, AA Kodama, O Berwanger, B Weber, LH Mota

**Affiliations:** 1 Research Institute - Hospital do Coração, São Paulo, Brazil

## Introduction

Our objective was to evaluate if the implementation of a multifaceted intervention program for quality improvement in ICUs of nonacademic hospitals would decrease mortality and the catheter-related bloodstream infection rate (CRBSI).

## Methods

A clinical practice improvement program involving 17 Brazilian ICUs of nonacademic public hospitals located far from major economic centers under coordination of a not-for-profit private hospital with the support of Brazilian Ministry of Health. We implemented the following interventions: (1) hospital visits to assess the facilities, human resources and processes; (2) workshop with hospital directors and ICU coordinators to elaborate improvement proposals based on the initial visit findings; (3) multidisciplinary videoconference lectures every 3 weeks about critical care medicine assistance and quality issues; (4) website containing project educational material, videoconference recordings, and an evidence-based practice course; (5) subscription of an electronic clinical information resource for all participating hospitals (UpToDate^®^); (6) 3-day workshop to share the coordinating institution quality improvement practices with directors and ICU coordinators from the 17 participant institutions; (7) 3-day nursing visits from the coordinating hospital to perform advice on care practice; (8) basic life support courses, 56 vacancies per hospital, and fundamentals of critical care support, 30 vacancies; and (9) implementation of a web-based system to collect ICU and hospital mortality, SAPS3, standardized-mortality ratio (SMR) and CRBSI after June 2011. We assessed variation of SMR and CRBSI on time using weighted linear regression, and variation of mortality on time using generalized-estimating equations.

## Results

The results are presented in Table 1.

**Table 1:** 

Outcome	Month					*P *value
	
	June	July	August	September	Odds ratio (95% CI)	
ICU death, *n *(%)	100 (36.0%)	122 (38.5%)	137 (34.9%)	82 (24.9%)	0.83 (0.75; 0.93)	0.001
In-hospital death, *n *(%)	108 (38.6%)	125 (43.0%)	151 (38.5%)	96 (29.2%)	0.85 (0.76; 0.94)	0.002

					**Mean difference (95% CI)**	

SMR, mean (SD)	1.25 (0.37)	1.63 (0.95)	1.31 (0.62)	0.89 (0.51)	-0.15 (-0.28; -0.01)	0.04
CRBSI, mean (SD)	11.8 (19.7)	4.3 (11.9)	4.4 (5.4)	3.3 (6.0)	-2.11 (-4.68; 0.45)	0.10

## Conclusion

A multifaceted intervention program applied to a network of ICUs in nonacademic public hospitals reduced mortality.

